# Association of N6-methyladenosine with viruses and related diseases

**DOI:** 10.1186/s12985-019-1236-3

**Published:** 2019-11-11

**Authors:** Fang Wu, Wenzhao Cheng, Feiyuan Zhao, Mingqing Tang, Yong Diao, Ruian Xu

**Affiliations:** 1 Engineering Research Center of Molecular Medicine, Ministry of Education, Huaqiao University, Xiamen, China; 2 School of Medicine, Huaqiao University, Xiamen, China; 3 Stem Cell Laboratory, The Second Affiliated Hospital, Fujian Medical University, Quanzhou, China; 4 Fujian Provincial Key Laboratory of Molecular Medicine & Fujian Provincial Key Laboratory of Precision Medicine and Molecular Detection in Universities, Xiamen, China

**Keywords:** N6-methyladenosine (m6A), Methyltransferases, Demethylases, m6A-binding proteins, Viral diseases

## Abstract

**Background:**

N6-methyladenosine (m6A) modification is the most prevalent internal modification of eukaryotic mRNA modulating gene expression. m6A modification is a dynamic reversible process regulated by three protein groups: methyltransferases (writers), demethylases (erasers), and m6A-binding proteins (readers). m6A modification is involved in all phases of RNA metabolism, including RNA folding, stability, splicing, nuclear exporting, translational modulation and degradation.

**Main body:**

In recent years, numerous studies have reported that abnormal m6A modification causes aberrant expression of important viral genes. Herein, we review the role of m6A in viral lifecycle and its contribution to the pathogenesis of human diseases. Particularly, we focus on the viruses associated with human diseases such as HIV-1, IAV, HBV, HCV, EBV and many others.

**Conclusions:**

A better understanding of m6A-virus relationship would provide new insights into the viral replication process and pathogenesis of human diseases caused by viruses. In addition, exploration of the role of m6A in disease-causing viruses will reveal novel approaches for the treatment of such diseases.

## Background

Like histone and DNA, RNA undergoes covalent modifications that fine-tunes its function [1]. Post-transcriptional modification of RNA is very common in eukaryotes, with more than one hundred types of RNA modification reported so far [2]. Most of these modifications occur on ribosomal RNA (rRNA) and transfer RNA (tRNA), which modulate RNA structures, functions, translation, as these RNAs are accessory molecules in eukaryotic processes [2, 3]. Messenger RNA (mRNA), a primary information-bearing molecule, is also post-transcriptionally modified [2]. As early as 1970s, researchers discovered that N6-methyladenosine (m6A) is present on cellular mRNA [4]. Since then, m6A modification has been identified in many mRNA, with 25% of all cellular mRNAs containing multiple m6A residues [5, 6]. Furthermore, it has been found that m6A modification mostly occur at RRACH motif (R = A or G, H = A, C, or U), and m6A sites are significantly clustered around transcription start sites, exonic regions flanking splicing sites, stop codons, 5’untranslated region (5’UTR) and 3’untranslated region (3’UTR) [7–9].

m6A modification is dynamically and reversibly regulated by methyltransferases (writers) [10, 11], and removed by demethylases (erasers) [12]; in addition, it functions by interacting with m6A binding proteins (readers), or by indirectly altering the structure of modified RNA to regulate RNA reader–protein interactions [6]. It has been demonstrated that m6A modification and its related enzymes play an important role in different phases of mRNA life [13].

Writers induce m6A modification, which is mediated by methyltransferase-like 3 (METTL3), methyltransferase-like 14 (METTL14), Wilms’ tumor 1-associating protein (WTAP), methyltransferase-like 16 (METTL16), RNA binding motif protein 15 (RBM15), and KIAA1429 [10, 14–16]. Bokar et al. discovered a ~ 200 kDa methyltransferase (MT) complex in nuclear extract lysate from HeLa cell which exhibited methyltransferase activity, from which a 70 kDa protein was identified, named MT-A70 or METTL3 [17]. Recently, *Liu* et al. found that METTL14, an RNA writer, forms a stable heterodimer core complex with METTL3 [10]. This complex component facilitates m6A deposition on nuclear RNA in mammalian cells. Furthermore, the combination of METTL3 and METTL14 dramatically enhanced methyltransferase activity compared to single protein. In addition, WTAP is a regulatory subunit in the m6A methyltransferase complex. Although it lacked methylation activity, it interacted with METTL3/14 complex to influence the deposition of m6A. Moreover, Ping and colleagues showed that WTAP might play a critical role in epitranscriptomic regulation of RNA metabolism [11]. Besides WTAP, RBM15 and KIAA1429, which are part of the complex, affected the activity of METTL3/14 [18]. Schwartz et al. showed that depletion of KIAA1429 decreased m6A level in vitro [19]. Moreover, knockdown of RBM15 caused a significant reduction of m6A deposition on mRNAs [15]. This means RBM15 and KIAA1429 might be involved in methylation process. A recent study [14] identified a new component of writers METTL16, which is independent from the METTL3/METTL14/WTAP complex, that caused m6A deposition on U6 snRNA [20] and U6-like hairpins of MAT2A mRNA [21] in a C-m6A-G context.

It has been demonstrated that m6A modification on RNA can be removed by at least two erasers, alkB homologue 5 (ALKBH5) or the fat mass and obesity-associated (FTO) proteins. Generally, ALKBH5 and FTO are mainly localized in the nuclear compartment [12, 16]. FTO is homologous to the DNA repair protein AlkB, and is involved in oxidative demethylation of 3-methylthymine in single-stranded DNA and in the 3-methyluracil activity in single-stranded RNA [22]. Several studies have reported that FTO is a potent regulator of nuclear mRNA processing events, participating in alternative splicing and processing of the 3′ end mRNA [23]. Additionally, as a demethylase, FTO manipulates the level of N6,2′-O-dimethyladenosine (m6Am); as depletion or overexpression of FTO selectively regulated the abundance of mRNAs containing m6Am in cells. Moreover, FTO reduced the stability of m6Am mRNAs, thus preferentially demethylated m6Am rather than m6A [24]. Zheng et al. demonstrated that ALKBH5 contributed to the removal of m6A modification from nuclear RNA (mostly mRNA) both in vitro and in vivo [16]. Furthermore, ALKBH5 significantly regulated the nuclear RNA export, metabolism and gene expression, indicating that reversible m6A modification on RNA has broad biological effects.

**Fig. 1:**
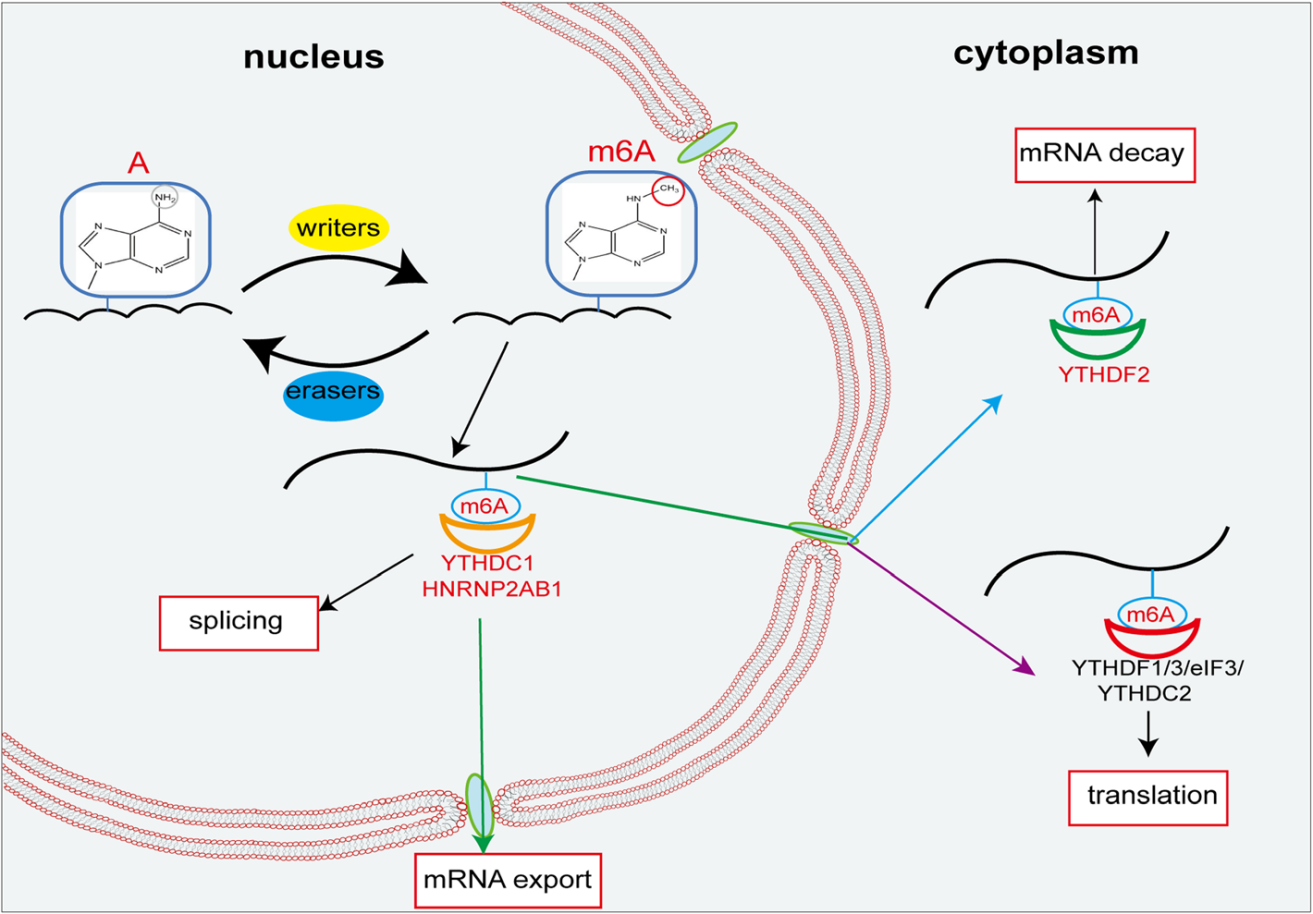
A schematic model that illustrates the roles and regulation of m6A modification. m6A modification is regulated by “writers” “erasers” and “readers”. Writers and erasers are mainly located in the nucleus, where they modulate mRNAs splicing and nuclear export. YTHDC1 and HNRP2AB1 as nuclear readers also play a vital role in nuclear export. In the cytoplasm, YTHDF2 promotes degradation of mRNA; whereas YTHDF1/3, YTHDC2 and eIF3 participate in translation

Proteins that selectively bind m6A sites are defined as m6A “readers”, which exert regulation by influencing the recognition of methylated RNA. It has been established that the YTH N6-methyladenosine RNA-binding protein family comprising YTHDF1, YTHDF2, YTHDF3, YTHDC1, and YTHDC2 is the major protein family among all “readers”. In general, YTHDF1 directly enhanced translation through binding with the m6A modification in 3’UTR region [25]; YTHDF2 recruited CCR4-NOT de-adenosine complexes to promote mRNA decay [26]; YTHDF3 acted as a helper of YTHDF1 and YTHDF2 [27, 28]. YTHDF1, 2, and 3 are dominantly located in the cytoplasm, while YTHDC1 is mainly localized in the nucleus where it regulates mRNA export from nucleus to cytoplasm and induce exon inclusion [29, 30]. YTHDC2 modulates the translation of RNA by interacting with the 40-80S subunit in the cytoplasm [31]. Recently, Huang et al. identified a new family- the insulin-like growth factor 2 mRNA-binding proteins (IGF2BPs, including IGF2BP1/2/3) [32]. In contrast to YTH domain family, IGF2BPs bind to m6A-modified mRNAs through GG(m6A)C, a typical m6A motif, to promote mRNA stability. Moreover, other proteins have been found to recognize m6A, such as eIF3 and HNRNP2AB1. eIF3, as part of the 43S pre-translational initiation complex, promotes protein translation by binding to the m6A site of mRNA 5’UTR region [24, 33, 34]. Therefore, m6A is dynamically regulated by writers, erasers, readers and other potential proteins associated with these regulators (Fig. 1).

## Main text

Accumulating evidence shows that m6A modification not only exists in eukaryotic cells but also in viruses. Currently, it is known that deregulation of m6A modification is associated with diseases caused by pathogenic viruses. m6A has long been identified in RNA transcripts of viruses and it regulates nuclear replication in viruses such as influenza A virus, simian virus 40, Rous sarcoma virus, avian sarcoma virus, and adenovirus [35–38]. In recent years, numerous studies have revealed that m6A modification regulates viral life cycles and m6A modification in pathogenic viruses is increasingly being investigated. Other scholars have revealed that m6A modification influences the expression of key genes involved in the viral life. In addition, m6A modification plays a role in the inhibition or promotion of different types of pathogenic viruses (Table 1). Therefore, exploring the biological functions of m6A methylation in different viruses is of great significance to understanding the pathogenesis and innovative prevention of viral infection. Relationships between m6A and viruses, in relation to diseases caused by different types of viruses are reviewed in following sections.

**Table 1:** The regulators of m6A in different viruses

Virus/cancer type	Molecule	Change	Sample source	Target	Biological function	Reference
HIV	METTL3/METTL14	Down	CD4^+^T cell	Rev	Suppress virus replication	[39]
	ALKBH5	Down	CD4^+^T cell	Rev	Promote virus replication	
	YTHDF1–3	Up	CEM-SS T cell	–	Promote virus replication	[1, 39]
			HeLa/CD4 cell	Gag	Suppress virus replication	[40]
EV71	METTL3	Down	Vero cells	RdRp 3D	Decrease in virus titer	[41]
	FTO	Down	Vero cells	–	Promote in virus titer	
	YTHDF2/3	Down	Vero cells	–	Suppress virus replication	
IAV	METTL3	Down	A549 cell	–	Suppress IAV replication	[42]
	YTHDF2	Up	A549 cell	–	Promote IAV replication	
KSHV	METTL3	Down	BCBL1 cell	ORF50	Suppress viral lytic replication	[43]
	FTO	Down	BCBL1 cell	ORF50	Promote viral lytic replication	
	YTHDC1	Up	BCBL1 cell	ORF50	Promote ORF50 pre-mRNA splicing	
	YTHDF2	Down	iSLK.219/ iSLK.BAC16 cell	ORF50	Suppress transcription in ORF50	[44]
HBV	METTL3/14	Down	HepAD38 cell	–	Promote HBc/s protein expression and the half-life of pgRNA	[45]
	FTO/ALKBH5/YTHDF2–3	Down	HepAD38 cell	–	Suppress HBc/s protein expression and the half-life of pgRNA	
HCV	METTL3/14	Down	Huh7 cell	–	Enhance titer of HCV	[46]
	FTO	Down	Huh7 cell	–	Decrease viral titer	
	YTHDF1–3	Up	Huh7 cell	–	Suppress HCV replication	
HCC	METTL3	Down	Patient sample/ HepG2, Huh-7 and MHCC97L	SOCS2	Attenuate SOCS2 mRNA stability	[47]
	METTL14	Down	Patient sample/ tumor tissues/ SMMC-7721, Hep3B and HepG2	miR-126	Regulate processing of miR-126 by DGCR8	[48]
CC	FTO	Up	Patient sample/ SiHa cell	–	Induce poor prognosis	[49, 50]
ZIKV	METTL3/14	Down	Vero cell	–	Increase viral titer	[51]
	ALKBH5/FTO	Down	Vero cell	–	Decrease viral titer	
	YTHDF2	Up	Vero cell	–	Suppress ZIKV replication	
SV40	METTL3	Down	BCS40 cell	–	Reduce SV40 replication	[52]
	YTHDF2	Up	BCS40 cell	–	Enhance replication of SV40	
EBV	METTL14	Up	LcLs and Akata cells	EBNA3C	Promote growth and proliferation of EBV-infected cells	[53]

### Human immunodeficiency virus I related diseases

Human immunodeficiency virus I (HIV-1) is the prototype member of the retroviral lentiviruses family and is the etiologic agent of acquired immunodeficiency syndrome (AIDS). People infected with HIV-1 require long-term antiviral treatment, and interruption of the treatment triggers rapid rebound viremia [54]. Therapies for viral infections include vaccines and other drugs. So far, no completely effective treatments have not been developed. Further investigations are required to seek more effective and safe treatments for this disease.

Investigations into m6A modification on HIV have lagged behind other known viruses such as influenza virus, adenovirus, Rous sarcoma virus, and simian virus 40 for almost 40 years [35–38].Recently, Lichinchi et al. reported the presence of m6A modification on HIV-1 RNA, and characterized the molecular features, topology and function of the viral-host RNA m6A during CD4^+^T cell infection [39]. They used HIV infected CD4^+^T cells as experimental subjects to analyze their RNA by methylated RNA immunoprecipitation sequencing (MeRIP-seq). Data showed that HIV-1 infection increased m6A modification level both in host and viral mRNAs compared with the un-infected cells. Knockdown of METTL3/14 suppressed virus replication, while knocking down of ALKBH5 produced opposite results. This indicates that m6A promotes replication of HIV. Subsequently, they discovered that m6A enrichment region was mainly located at the 5’UTR and 3’UTR of HIV genomic RNA, this finding was consistent with that of Tirumuru et al (their RNA samples were isolated from T-cells or primary CD4^+^T-cells infected with replication-competent HIV-1_NL4–3_ and analyzed with high-throughput RNA sequencing (m6A-seq)) [40]. However, Kennedy et al. used photo-crosslinking-assisted m6A sequencing (PA-m6A-seq) technique to analyze the RNA samples isolated from infected human CD4^+^CEM-SS T-cells. They found the enrichment of m6A was merely located at the 3′UTR of HIV-genomic RNA [1]. The discrepancies between these studies might stem from the different mapping techniques used or cell types used for RNA isolation.

Besides writers and erasers, the studies mentioned above revealed that three YTHDF proteins could interact with HIV methylated RNA, but only two of these promoted HIV replication [1, 39]. Kennedy et al. confirmed that overexpression of YTHDF1/3 enhanced the expression of HIV-1 at mRNA and protein level in HIV infected HEK-293 T cells, whereas YTHDF2 did not influence viral replication and protein expression in CEM-SS T cells. In contrast, Tirumuru et al. found that YTHDFs inhabits HIV replication. The mechanisms for the differences among these studies warrants further research. Elsewhere, Lichinchi et al. confirmed that m6A modification existed in 56 host genes from infected T cells using MeRIP-seq. The 56 host genes were involved in viral replication, implying that m6A modification may enhance viral replication by regulating the expression of the 56 host genes.

Rev response element (RRE) is a well-characterized structural and functional RNA element within HIV-1 env gene. After translation, Rev. proteins are imported back into the nucleus where they assemble at the RRE to form an active nuclear export complex to facilitate the transit of viral transcripts into cytoplasm [55]. This process is an essential step for viral replication. Lichinchi et al. found that m6A modification on viral RNA positively affected the interaction between HIV Rev. protein and RRE RNA. This promoted the formation of Rev–RRE complexes and the nuclear export of viral RNA, and hence viral replication. Overall, the links between m6A-related proteins and HIV-1 need to be clarified to identify new mechanisms modulating HIV-1 replication and interaction with the host immune system.

### Enterovirus 71 related diseases

Enterovirus 71 (EV71) is a single-stranded retrovirus with a genome of approximately 7.5 kb. EV71 belongs to the genus enterovirus in the Picornaviridae family, which contains three genotypes (A, B and C) and other sub-genotypes. EV71 infections are common in young children, and is one of the etiologic agents of hand, foot, and mouth disease (HFMD) characterized with fever, sores in mouth and a rash with blisters [56]. Since the discovery of EV71 in 1969, innumerable outbreaks and epidemics have been reported worldwide, especially in Asia and Pacific regions, such as China, Korea, Singapore, Japan and Vietnam [57]. Unfortunately, there are no effective antiviral drugs for diseases caused by EV71 infections.

With the rapid development of sequencing technology, several discoveries have been made. For instance, Hao et al. found that EV71 RNA also contains m6A modification in the coding region of VP, 3D and 2C. Specifically, they found that the expression of METTL3, METTL14, YTHDF1–3 and YTHDC1 was increased when the host was infected with EV71, whereas the expression of FTO was decreased [41]. In addition, the expression of m6A regulatory gene affected viral replication. Similarly, mutation in m6A modification sites were found to decrease the production of EV71 progeny virus and protein expression in the infectious clones. Moreover, METTL3 silencing decreased the virus titers and RNA copies, but knockdown of FTO produced opposite results. These results indicate that METTL3 and FTO affect the replication of EV71, suggesting the m6A modification may positively regulate EV71 lifecycle. Meanwhile, knockdown of YTHDF2 and YTHDF3 in Vero cells inhibited viral replication. A co-immunoprecipitation (co-IP) experiment revealed that METTL3 could interact with viral RNA-dependent RNA polymerase (RdRp) 3D, and overexpression of METTL3 increased this protein and contributed to sumoylation and ubiquitination of the 3D polymerase during viral replication. METTL3 functioned as a positive regulator of EV71 replication in the report by Hao and colleagues. Whether METTL3 should be used as a new therapeutic target remains to be elucidated.

### Influenza a virus related diseases

Influenza A virus (IAV) is a major cause of upper and lower respiratory tract infections, posing a huge threat to human health. Currently, influenza prevention and treatment strategies include annual vaccination and antiviral drugs [58]. However, frequent antigens variation on viral surface was caused by antigenic shift and drift, allowing influenza viruses to escape antibody-mediated immunity following vaccination [59]. Therefore, it is important to explore new anti-viral drugs to replace the traditional methods for effective control of influenza virus diseases. Previously, it was demonstrated that the influenza virus mRNAs contain internal m6Amodification that is required for viral replication [38]. Courtney et al. reported that m6A modification affected the viral replication and gene expression of IAV [42]. In detail, they used CRISPR/Cas9 system or 3-deazaadenosine (3-DAA), an inhibitor of m6A modification which inhibits SAM, to suppress the expression of METTL3 in A549 cells. They found that the viral replication and mRNA level of IAV were significantly reduced. Conversely, the expression of YTHDF2 promoted IAV replication and increased the number of infectious particles in A549 cells, whereas YTHDF1 and YTHDF3 did not affect on IAV replication. These results indicate that m6A modification might play a critical role in promoting the replication of IAV. Later, it was found that m6A modification not only exists on the positive strands of IAV mRNA/cRNA and the negative strands of vRNA, but it is also present on the viral mRNAs of HA, NA, M1/M2 and NP as determined by PAR-CLIP and PA-m6A-seq sequencing. Finally, Courtney et al. silenced m6A sites in hemagglutinin segment RNA, and found that the expression of hemagglutinin and viral replication, as well as the pathogenicity of IAV were reduced in mice.

Collectively, these findings imply that m6A modification might play a critical role in viral replication, transcription, and production. Thus, these findings add to our understanding of m6A modification in IAV.

### Kaposi’s sarcoma-associated herpesvirus related diseases

The Herpesviridae family is one of the most prevalent human pathogens, and 90% of adults are infected by at least one of the eight herpesvirus subtypes [60]. KSHV is the causative agent for multiple malignancies including Kaposi’s sarcoma (KS), primary effusion lymphoma (PEL) and multicentric Castleman’s disease (MCD) [61–63]. Latency is common among all herpes viruses. Recently, Ye et al. discovered that reactivation of KSHV would be stalled when the newly transcribed viral RNAs fail to undergo post-transcriptional modification with m6A [43]. Reactivation of the latent virus, resets the epigenetic processes, leading to the transactivation of viral genome [64]. Therefore, a better understanding of the relationship between KSHV and m6A is a prerequisite for developing prevention and treatment strategies for KSHV-induced diseases.

Ye et al. reported that the level of m6A-modified mRNA (m6A-mRNA) for a given viral transcript increased substantially when infected cells were stimulated to undergo lytic replication [43], and this was also observed by Hesser et al. in vitro [44]. KSHV replication transcription activator (RTA), an immediate early protein encoded by Open reading frame 50 (ORF50), played an essential role in KSHV lytic replication and inhibited virion production. Ye et al. [53]stimulated KSHV-infected cells to undergo lytic replication, and observed that the splicing of pre-mRNA was inhibited in the presence of 3-DAA. Moreover, they showed that knockdown of METTL3 or inhibition of methyltransferase complexes with 3-DAA suppressed viral lytic replication in BCBL1 cells. On the other hand, knockdown of FTO moderately increased viral lytic replication. These observations indicate that KSHV activates the expression of viral genes and the lytic cycle through manipulating the host m6A modification machinery. It has also been reported that YTHDC1 interacts with the splicing factors SRSF3 and SRSF10 to form a ternary complex, and can bind to the ORF50 pre-mRNA to promote its expression. Moreover, the expression of RTA increased the modification level of m6A, further inducing the splicing of pre-mRNA.

In KSHV iSLK.219 and iSLK.BAC16 reactivation models, Hesser et al. [44] discovered that YTHDF2 plays an important role in the replication of KSHV. They used si-RNA to knock down the expression of METTL3 and YTHDF2, and found that few virions were produced after inhibition of METTL3, while knockdown of YTHDF2 displayed almost no virions. Subsequent experiments from the same group showed that inhibition of YTHDF2 affected the expression of immediate early lytic gene and viral activation. ORF50 acted as a viral transcription trans-activator which played a critical role in the lytic induction of KSHV in response to all known stimuli [65]. They also revealed that m6A modification regulated the expression of ORF50 in iSLK.219 cells, and YTHDF2 deletion impaired the transcription of ORF50. This finding suggests that YTHDF2 have a pro-viral role in DNA and RNA viruses. Besides, Tan et al. [66] found that m6Am modifications occur in KSHV transcripts during latent and lytic replication. Moreover, YTHDF2 in m6Am negatively affected lytic replication by decaying transcripts of KSHV. These observations illustrate that YTHDC1 and YTHDF2 might be involved in KSHV lifecycle, suggesting that the regulation of ORF50 expression through m6A modification could be a new research direction into the mechanisms of KSHV infections.

### Hepatitis virus related diseases

Hepatitis B virus (HBV) is a DNA virus belonging to the Hepadnaviridae family. HBV infection causes chronic hepatitis and increases the risk of developing cirrhosis and hepatocellular carcinoma [67]. Recently, researchers have demonstrated that the life cycle of HBV is influenced by m6A modification that can affect the expression of viral oncoprotein and the reverse transcription of pre-genomic RNA (pgRNA) [45]. Imam et al. found that m6A modification of HBV transcript negatively regulated the expression of HBV protein and the half-life of pgRNA. Knock down of METTL3 and METTL14 increased the expression of HBc, HBs and the half-life of pgRNA, similar to YTHDF2 and YTHDF3. In contrast, low expression of FTO and ALKBH5 produced opposite results.

Apart from HBV, Hepatitis C virus (HCV) RNA also harbors m6A modification [46]. Knockdown of METTL3 and METTL14 expression enhanced the titer of HCV, whereas FTO inhibition decreased the viral titer. They also found that YTHDF protein played a negative role in HCV replication. In addition, these proteins compete with the core protein of HCV by preferentially binding to the *Env* to inhibit the packaging of viral RNA.

Chronic infection with HBV and HCV is the most cause of hepatocellular carcinoma (HCC) [45, 68]. Accumulating evidence indicates that HBV and HCV together with HCC oncoproteins are regulated by m6A modification. Recently, two studies revealed that m6A modification is involved the progression of HCC via METTL3 and METTL14. Chen et al. discovered that METTL3 which is highly expressed in HCC promoted the cellular proliferation, migration and colony formation of HCC cells in vitro, and enhanced tumorigenicity in vivo [47]. Suppressors of cytokine signaling 2 (SOCS2), a target gene of METTL3-mediated m6A modification, inhibit various types of cancers. Chen et al. found that knockdown of METTL3 inhibited the transcription of SOCS2 mRNA. They also demonstrated that the effect of METTL3 on SOCS2 was mediated by m6A “reader” YTHDF2. Moreover, low expression of SOCS2 was significantly associated with poor overall survival and disease-free survival of HCC patients. Ma et al. reported that METTL14, which is significantly down-regulated in HCC, regulates HCC development [48]. Compared to the study by Chen [47], the levels expression of METTL3, Wilms tumor 1–associated protein, KIAA1429, and ALKBH5 in HCC were not significantly different [48].. METTL14 is associated with frequent recurrence and poor survival; and abnormal levels of METTL14 are related with tumor differentiation, tumor stage, tumor encapsulation, microsatellite and microvascular invasion. Ma et al. concluded that microRNA126 (miR126), a downstream target of METTL14, is regulated in an m6A-dependent manner. METTL14 regulates the maturation of pri-miR126 to miR126 by enhancing the recognition and binding of the microprocessor protein DGCR8 to pri-miRNA. Collectively, these studies reveal that m6A modification plays a pivotal role in HCC through different mechanisms mediated by various m6A related proteins.

Collectively, these results show that METTL3 and METTL14 modulate m6A modification during the development of HCC. Thus, SOCS2 and miR126 may be target genes for the treatment of HCC. Further studies are required to determine the implication of m6A modification in HBV, HCV and HCC.

### Human papillomavirus related diseases

Human papillomavirus (HPV) is a small DNA virus with a genome of approximately 8 kb belonging to Papillomavirus genus of the Papillomavirus family. Cervical cancer (CC) is the fourth most prevalent cancer in women and is one of the most common gynecological malignancies worldwide [69, 70]. Although significant advances have been made in cancer detection and treatment during the past few decades, the 5-year survival rate remains low. Thus, it is urgent to determine molecular mechanisms underlying the development of CC, and to explore innovative therapeutic strategies for this disease.

A recent study revealed that m6A methylation participates in the oncogenesis of cervical cancer. The study found that m6A plays a negative role in cellular proliferation tumor formation of CC [49]. Specifically, they found that the m6A level was significantly lower in cervical cancer tissues compared with adjacent non-cancerous tissues. Moreover, for patients with cervical cancer, the disease-free survival (DFS) and overall survival (OS) were significantly higher in patients with high m6A level than those with low m6A level, indicating that the level of m6A methylation could be a prognostic marker of cervical cancer. Knockdown of erasers (FTO and ALKBH5) or overexpression of writers (METTL3 and METTL14) suppressed the cellular proliferation and tumor formation of cervical cancer cells both in vitro and in vivo. Interestingly, Zhou et al. reported that overexpression of FTO induced chemo-radiotherapy resistance in cervical squamous cell carcinoma (CSCC), the major type of cervical cancer [50]. Unfortunately, FTO-mediated upregulation of β-catenin via mRNA demethylation triggered the activation of excision repair cross-complementation group 1 (ERCC1), further contributing to this resistance. It was also recognized that high expression level of FTO was associated with poor prognosis. Understanding the role of m6A in cervical cancer is expected to provide clues for optimal treatment of CC. So far, no study has described the relationship between HPV and m6A methylation, and future studies should address this gap.

### Zika virus related diseases

Zika virus (ZIKV), which causes Zika virus disease (ZVD)-a type of acute infectious disease, was firstly isolated from the serum of apyrexial rhesus monkey caged in the canopy of Zika Forest [71]. In 2007, a large outbreak of ZIKV occurred in Yap Island of the Western Pacific [72], and later on in French Polynesia in 2013 [73]. More recent outbreaks were reported in Brazil and South America which reached epidemic levels [74]. Currently, there are no specific antiviral drugs for ZIKV; therefore, it is imperative to explore innovative means to prevent ZIKA infections.

One study demonstrated the existence of m6A modification in ZIKV. Lichinchi et al. confirmed that ZIKV viral RNA was methylated and twelve discrete m6A peaks spanning the full length of ZIKV RNA were identified, most of which were present in the region encoding NS5 and the 3’UTR [51]. Like other virus, ZIKV viral-RNA is also modified at adenosines by writers and erasers; and perturbation of ZIKV m6A affects the replication efficiency and viral titer of ZIKV. Knockdown of writers (METTL3 or METTL14) significantly increased the viral-production, viral titer, ZIKV RNA levels in cell supernatants and the expression of ZIKV envelope protein, whereas silencing of erasers (ALKBH5 or FTO) produced opposite effect. It was also reported that readers (YTHDF proteins) that bind to ZIKV RNA may regulate the replication of ZIKV. Compared to YTHDF1 and YTHDF3, YTHDF2 has the greatest effect on ZIKV replication, RNA expression and stability of viral RNA. Generally, these results point to a new mechanism for the relationship between m6A associated enzymes and ZIKV. This knowledge is expected to generate ideas for the design of treatments for ZIKV-related diseases.

### Simian virus 40 related diseases

Simian Virus 40 (SV40), a polyomavirus of the rhesus macaque, is a double-stranded DNA virus, and a potent DNA tumor virus reported to induce human primary brain tumors, malignant mesotheliomas, bone cancers, and non-Hodgkin’s lymphoma [75, 76]. In 1979, it was reported that late SV40 16S and 19S mRNAs contain numerous m6A modified residues [35]. However, the exact location of m6A modified residues is not known, and the functional significance of these residues is unclear. Recently, Tsai et al. identified and precisely mapped the m6A peaks on SV40, including eleven peaks in late transcripts, and two in the early region by photo-crosslinking-assisted m6A sequencing (PA-m6A-seq), photoactivatable ribonucleoside-enhanced crosslinking and immunoprecipitation (PAR-CLIP) [52]. In addition, they demonstrated that overexpression of YTHDF2 significantly enhanced the replication of SV40, and mutation of YTHDF2 inhibited the replication of SV40. Furthermore, knockdown of YTHDF2 or METTL3 by gene editing suppressed the expression of SV40 structural gene in the permissive cell line BSC40. Overall, this investigation revealed that m6A modification plays a positive role in the regulation of SV40 life cycle.

### Epstein–Barr virus related diseases

Epstein–Barr virus (EBV), a herpesvirus, is an oncogenic virus first isolated and identified from a Burkitt’s lymphoma patient in 1964 [77]. As one of the most common human viruses, EBV causes infectious mononucleosis and is associated with specific forms of lymphoma. There are about 200,000 cases of cancer associated with this virus, with about 140,000 deaths reported annually [78]. Common antiviral therapies can suppress the replication of active-viruses. However, the existing vaccines or drugs are not effective in eradicating the latent infection of EBV.

To date, only one study has reported the association of m6A “writers” with EBV mainly through METTL14 [53]. METTL14 was increased in EBV latently infected cells and decreased during the lytic infection phase. It should be noted that METTL14 facilitates the cellular proliferation and colony formation of EBV transformed cells in vitro, and enhances the oncogenesis of EBV tumorigenicity in vivo. It was reported that METTL14 impairs the stability of the latent genes (such as EBNA1, EBNA3C, and LMP1) and lytic genes (such as BRLF1, gp350, and BMRF1). Elsewhere, EBNA3C was found to be a downstream target gene of METTL14, and EBV antigen up-regulated and stabilized METTL14. EBV latent antigens are the major contributors to EBV-associated malignancies, and low expression of EBV latent antigens attenuates the EBV-mediated tumorigenesis. Therefore, targeting METTL14 may be a key strategy for controlling EBV-associated cancer.

## Conclusions

The viruses described in this review are harmful to human health. Some viruses RNA undergo m6A modification such as Rous sarcoma virus (RSV) [79], vesicular stomatitis virus (VSV) [80], and adenoviruses [37], and are less pathogenic in human. Several advances have been made in understanding human pathogenic viruses in recent years, however, only few drugs for these diseases have been put on the market in the past decades. For this reason, it is necessary to focus on the development of new treatments that may replace or improve the standard therapies.

The emergence of posttranscriptional modification of RNAs, especially the methylation of RNAs, has triggered several investigations into the role of such modifications on gene expression, cell behaviors, and physiological conditions in many species, including humans. Among more than 100 kinds of chemical modifications, m6A is the most common modification initially discovered in the poly(A) RNA fractions and has been predicted to be involved in mRNA processing [4]. m6A is a dynamic and reversible process initiated by “writers” and removed by “erasers” and binds to “readers”. Emerging evidence indicates that aberrant expression of proteins related to m6A modification is associated with the development of various cancers, such as acute myeloid leukemia [81, 82], lung cancer [83], and hepatocellular carcinoma [47, 48]. Particularly, m6A has been detected in viral mRNA and the RNA of retroviruses. It can influence the expression of viral genes and viral life cycle, providing a new method for the treatment of many malignant virus-related diseases.

The development of small molecule inhibitors targeted at writers, erasers and readers in m6A modification process is expected to improve antiviral therapy. Recent studies have shown that the use of writer inhibitors may affect viral replication and the development of tumors [1, 47, 84]. Thus, inhibition of m6A modification may be a potential therapy for virus induced diseases.

Numerous studies have shown that m6A modification plays a significant role in human pathogenic viruses, but there are some challenges to be addressed. First, the specific mechanism explaining the interaction of m6A modification proteins with viral RNA is largely unknown. Second, before the m6A modification-related regulatory genes and proteins can be used as prognostic and diagnostic markers for some viral diseases, studies are needed to explore the specificity and targeting precision of these molecules. Moreover, the effects of these biomolecules on normal cells remains to be clarified. Third, many researchers have reported that substances similar to inhibitors could be used to block m6A-modified abnormalities to control related diseases, but due to the lack of large clinical trials, the corresponding effects of these substances are not fully known. Thus, further studies are required to address these issues.

## Data Availability

Not applicable.

## Abbreviations

3’UTR: 3’untranslated region
5’UTR: 5’untranslated region
AIDS: acquired immunodeficiency syndrome
ALKBH5: alkB homologue 5
CC: Cervical cancer
co-IP: co-immunoprecipitation
CSCC: cervical squamous cell carcinoma
DAA: 3-deazaadenosine
DFS: disease-free survival
EBV: Epstein–Barr virus
ERCC1: excision repair cross-complementation group 1
EV71: Enterovirus 71
FTO: fat mass and obesity-associated proteins
HBV: Hepatitis B virus
HCC: Hepatocellular Carcinoma
HCV: Hepatitis C virus
HIV-1: Human immunodeficiency virus I
HPV: Human papillomavirus
IAV: Influenza A virus
IGF2BPs: the insulin-like growth factor 2 mRNA-binding proteins
KS: Kaposi’s sarcoma
KSHV: Kaposi’s sarcoma-associated herpesvirus
m6A: N6-methyladenosine
m6Am: N6,2′-O-dimethyladenosine
m6A-seq: high-throughput RNA sequencing
MCD: multicentric Castleman’s disease
MeRIP-seq: methylated RNA immunoprecipitation sequening
METTL14: methyltransferase-like 14
METTL16: methyltransferase-like 16
METTL3: methyltransferase-like 3
miR126: microRNA126
mRNA: messenger RNA
MT: methyltransferase
ORF50: Open reading frame 50
OS: overall survival
PA-m6A-seq: photo-crosslinking-assisted m6A sequencing
PAR-CLIP: photoactivatable ribonucleoside-enhanced crosslinking and immunoprecipitation
PEL: primary effusion lymphoma
RBM15: RNA binding motif protein 15
RdRp: RNA-dependent RNA polymerase
RRE: Rev response element
rRNA: ribosomal RNA
RSV: Rous sarcoma virus
RTA: replication transcription activator
SOCS2: suppressors of cytokine signaling 2
SV40: Simian Virus 40
tRNA: transfer RNA
VSV: vesicular stomatitis virus
WTAP: Wilms’ tumor 1-associating protein
ZIKV: Zika virus
ZVD: Zika virus disease
